# Atrioventricular Synchrony Restoration Aided by a Temporary Permanent Pacemaker in Right Ventricular Infarction and Complete Heart Block

**DOI:** 10.7759/cureus.54631

**Published:** 2024-02-21

**Authors:** Mahati Dasari, Akil Sherif, Pramukh Arun Kumar, Pradnya Brijmohan Bhattad, Zeynep Yukselen, Ajay K Mishra, Luigi Pacifico, Eddison Ramsaran

**Affiliations:** 1 Internal Medicine, Saint Vincent Hospital, Worcester, USA; 2 Cardiology, Saint Vincent Hospital, UMass Chan Medical School, Worcester, USA; 3 Cardiovascular Medicine, Saint Vincent Hospital, UMass Chan Medical School, Worcester, USA; 4 Cardiovascular Medicine, Saint Vincent Hospital, Worcester, USA

**Keywords:** dual-chamber pacing, myocardial infarction, complete heart block, cardiogenic shock, temporary permanent pacemaker

## Abstract

Pacemakers are effective treatments for a variety of bradyarrhythmias. Cardiac pacemakers generally consist of a pulse generator and one or more leads. The conventional temporary transvenous ventricular cardiac pacemaker utilizing a passive fixation lead is commonly associated with multiple complications such as increased infection rate, lead dislodgement, venous thrombosis, longer duration of hospital stay, and atrioventricular (AV) dyssynchrony. On the other hand, temporary permanent pacemakers (TPPM) utilize active fixation leads; hence, they provide lower capture thresholds, reliable pacing, lower rates of displacement, and fewer pacemaker-related infections. Here, we present a case of TPPM aiding AV synchrony restoration in complete heart block accompanying right ventricular (RV) infarction with refractory cardiogenic shock. Pacemakers are effective treatments for a variety of bradyarrhythmias. Cardiac pacemakers generally consist of a pulse generator and one or more leads. We present a case of TPPM aiding AV synchrony restoration in complete heart block accompanying RV infarction with refractory cardiogenic shock. TPPM pacing is a safe and effective technique for temporary bridge pacing to prevent AV dyssynchrony in hemodynamically unstable patients with cardiogenic shock from RV infarction and complete heart block. It also hastens recovery compared to a traditional single-chamber temporary pacemaker.

## Introduction

The conventional temporary transvenous ventricular cardiac pacemaker utilizing a passive fixation lead is commonly used as a bridge to a permanent pacemaker or sinus recovery in life-threatening bradycardia. However, it is associated with an increased infection rate, lead dislodgement, venous thrombosis, longer duration of hospital stay, and atrioventricular (AV) dyssynchrony [1]. Temporary permanent pacemakers (TPPMs) with bipolar active fixation leads and epicutaneous pulse generators are effective for prolonged quick pacing while bridging to permanent system implantation or recovery. TPPMs use a screw-in mechanism, allowing for durable attachment and stable pacing; hence, they provide lower capture thresholds, reliable pacing, lower rates of displacement, and fewer pacemaker-related infections [2]. Here, we present a case of TPPM aiding AV synchrony restoration in complete heart block accompanying right ventricular (RV) infarction with refractory cardiogenic shock.

## Case presentation

A 68-year-old male with known coronary artery disease (CAD) and mid-ascending aortic aneurysm presented with sudden-onset exertional chest pain, which developed while shoveling snow, midsternal, nonradiating, 7/10 in intensity, not relieved with rest or nitroglycerin, and associated with progressive shortness of breath and presyncope symptoms. He was hypotensive on presentation with a blood pressure (BP) of 75/47 mmHg.

**Figure 1:**
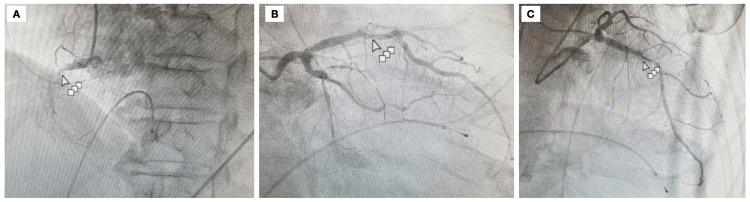
Cardiac catheterization showing (A) left anterior oblique straight view showing proximally occluded right coronary artery with a distal vessel filling with grade 2 left to right collaterals, (B) right anterior oblique cranial view showing 70-80% stenosis in the mid-segment of LAD, and (C) right anterior oblique caudal view demonstrating a lesion in the mid-segment of LAD.

Troponin was elevated to 9.73 ng/mL, over 100 times the standard upper limit of our laboratory reference range. An electrocardiogram (ECG) revealed a complete heart block and an evolving inferior wall infarction with inferior wall Q waves and ST segment elevation. A transthoracic echocardiogram revealed reduced left ventricular systolic function with an ejection fraction of 30%, inferoseptal and anteroseptal akinesis, and decreased RV function. A diagnostic coronary angiogram showed the left main free of CAD, left anterior descending (LAD) with 70-80% stenosis in the mid-segment, first obtuse marginal with 40% proximal stenosis, and right coronary artery (RCA) occluded proximally (Figure 1).

We did not intervene on the RCA at this time, as he had no further chest pain and had grade 2 collaterals to the distal RCA from the left system and Q waves on the ECG, all suggesting that occlusion had occurred over 24 hours ago. Additionally, the RCA was heavily calcified, and the creatinine was 4.1, raising the concern for contrast-induced nephropathy should PCI be attempted. Heparin drip, statin, and aspirin were initiated.

**Figure 2:**
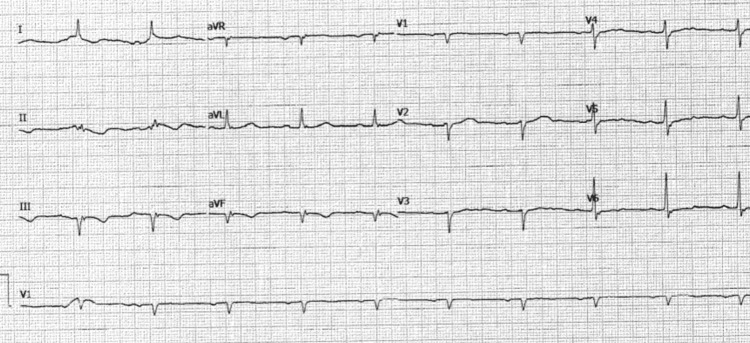
ECG showing normal sinus rhythm with pathological Q waves in inferior leads.

A single lead temporary ventricular pacemaker was placed for the complete heart block. He received 3.5 L of intravenous fluids, followed by norepinephrine initiation for BP support. An intra-aortic balloon pump (IABP) was placed for further hemodynamic support because of persistent hypotension during coronary catheterization on day one. His course was complicated with cardiac arrest secondary to ischemia-induced polymorphic ventricular tachycardia, reverting to a paced rhythm after three shocks. He depended on multiple vasopressors and an IABP to maintain a systolic BP over 90 mmHg. Consideration of coronary artery bypass grafting was declined, given a prohibitively high surgical risk. A mid-LAD PCI was performed to improve hemodynamics and facilitate safely weaning hemodynamic support. On day four, he underwent placement of a dual-chamber TPPM via subclavian access to provide AV synchrony to possibly increase his cardiac output and improve hemodynamics, RV function, and conduction system. After TPPM implantation, his hemodynamics immediately improved; hence, an IABP was removed. This was evidenced by the norepinephrine requirement at 22 mcg/min on day four, weaned to 4 mcg/min on day five. His pressor support was weaned entirely off by day six. On day 14 of hospitalization, he reverted to normal sinus rhythm (Figure 2).

The TPPM was removed on day 18 as he remained in normal sinus rhythm, not requiring further pacing. Subsequently, he was discharged on guideline-directed medical therapy (GDMT).

At six months, follow-up ECG showed normal sinus rhythm with Q-waves in inferior leads, and a transthoracic echocardiogram revealed an improved ejection fraction of 40-45%. At the one-year follow-up, he remained on appropriate GDMT and had fully recovered.

## Discussion

TPPMs play an essential role in many clinical situations requiring prolonged pacing, failure of conventional transvenous pacing, lead extraction procedures, or pacing device-related infections [3], or, as described in our case, hypotension and shock in the setting of bradyarrhythmias. TPPM placement requires an operator trained in placing active fixation leads under fluoroscopy guidance in the cardiac catheterization laboratory with a duration similar to permanent pacemaker implantation.

In most settings of complete heart block, single-chamber RV pacing serves as an adequate bridge to a permanent pacemaker or sinus recovery. However, our patient showed features of RV failure following the inferior infarction. In these situations, the systolic atrial output may become significant in addition to maintaining adequate preload. Furthermore, the presence of an RV pacemaker voided the atrial “kick” in the setting of AV dyssynchrony, leading to further hemodynamic compromise. Therefore, the placement of synchronized AV pacing was aimed at improving synchrony with the help of a TPPM system. However, based on the current literature review, only one published study has reported using a TPPM to promote AV synchrony in patients with sepsis and hemodynamically significant AV block with heart failure [4].

Lead dislodgement rates in conventional systems are reported in the literature to range from 17% [5] to as high as 30% [6], which reduces with active fixation leads in TPPM with reported dislodgement rates of 1.7% [2,7]. TPPMs also allow for extended durations of temporary pacing, especially in cases where a permanent pacemaker is not indicated [8].

Chihrin et al. have shown that using a TPPM is inexpensive compared to using a traditional temporary pacing system [9]. Additionally, active fixation leads allow better ambulation, thereby preventing muscle loss and deconditioning. This also reduces venous thrombosis rates and increases patient comfort [10]. There have also been reports of patients discharged from the hospital with TPPMs while awaiting complete recovery [7].

## Conclusions

A TPPM is an efficacious strategy instead of conventional temporary pacing systems as a temporary bridge to recovery in unstable patients with a potential for complete recovery such as in our case.

TPPM pacing is a safe and effective technique for temporary bridge pacing to prevent AV dyssynchrony in hemodynamically unstable patients with cardiogenic shock from RV infarction and complete heart block. Moreover, opting for dual-chamber pacemakers over traditional single-chamber temporary pacemakers to prevent AV dyssynchrony hastens recovery when TPPM is chosen as a bridge to the recovery strategy.
